# Zn^2+^ chelation by serum albumin improves hexameric Zn^2+^-insulin dissociation into monomers after exocytosis

**DOI:** 10.1371/journal.pone.0187547

**Published:** 2017-11-03

**Authors:** José A. G. Pertusa, Trinidad León-Quinto, Genoveva Berná, Juan R. Tejedo, Abdelkrim Hmadcha, Francisco J. Bedoya, Franz Martín, Bernat Soria

**Affiliations:** 1 Instituto de Bioingeniería, Universidad Miguel Hernández de Elche, Alicante, Spain; 2 Department of Cell Regeneration and Advanced Therapies, Andalusian Center of Molecular Biology and Regenerative Medicine-CABIMER, University of Pablo de Olavide-University of Sevilla-CSIC, Seville, Spain; 3 Biomedical Research Network on Diabetes and Related Metabolic Diseases-CIBERDEM, Instituto de Salud Carlos III, Madrid, Spain; 4 Department of Molecular Biology and Biochemistry Engineering, University Pablo de Olavide (UPO), Seville, Spain; Universidad Miguel Hernandez de Elche, SPAIN

## Abstract

β-cells release hexameric Zn^2+^-insulin into the extracellular space, but monomeric Zn^2+^-free insulin appears to be the only biologically active form. The mechanisms implicated in dissociation of the hexamer remain unclear, but they seem to be Zn^2+^ concentration-dependent. In this study, we investigate the influence of albumin binding to Zn^2+^ on Zn^2+^-insulin dissociation into Zn^2+^-free insulin and its physiological, methodological and therapeutic relevance. Glucose and K^+^-induced insulin release were analyzed in isolated mouse islets by static incubation and perifusion experiments in the presence and absence of albumin and Zn^2+^ chelators. Insulin tolerance tests were performed in rats using different insulin solutions with and without Zn^2+^ and/or albumin. Albumin-free buffer does not alter quantification by RIA of Zn^2+^-free insulin but strongly affects RIA measurements of Zn^2+^-insulin. In contrast, accurate determination of Zn^2+^-insulin was obtained only when bovine serum albumin or Zn^2+^ chelators were present in the assay buffer solution. Albumin and Zn^2+^ chelators do not modify insulin release but do affect insulin determination. Preincubation with albumin or Zn^2+^ chelators promotes the conversion of “slow” Zn^2+^-insulin into “fast” insulin. Consequently, insulin diffusion from large islets is ameliorated in the presence of Zn^2+^ chelators. These observations support the notion that the Zn^2+^-binding properties of albumin improve the dissociation of Zn^2+^-insulin into subunits after exocytosis, which may be useful in insulin determination, insulin pharmacokinetic assays and islet transplantation.

## Introduction

The insulin concentration in blood is approximately 1 ng/ml, and the predominant form is monomeric [1]. However, the insulin molecule associates into hexamers at higher insulin concentrations, and this feature is favored by acid or neutral pH and Zn^2+^ [2–4]. In β-cells, insulin is stored in the secretory granules as hexamers coordinated with two Zn^2+^ ions per hexamer [3]. In response to a stimulus, such as a rise of blood glucose or an increase in extracellular K^+^ concentration, β-cells release the hexameric Zn^2+^-insulin to the extracellular space [5]. However, *in vivo* experiments show that insulin is available as monomer within a few seconds of entering the portal vein [6]. In addition, it is generally accepted that the biologically active form of insulin is the monomer and not the hexamer. The reasons for this are i) the monomer is the predominant form in the bloodstream, ii) the majority of the receptor binding region is located at the dimer-forming surface [7,8] and iii) the insulin hexamer seems to be ineffective at activating the insulin receptor [9,10]. Thus, the physiological process of hexamer dissociation is a very important but poorly understood step in the regulation of insulin availability and glucose homeostasis by the organism.

It is established that at pH 7.3, Zn^2+^ coordination prevents hexamer dissociation into monomers. Thus, Zn^2+^ dilution and pH changes have been proposed as mechanisms to facilitate Zn^2+^-insulin hexamer disintegration [11]. However, micromolar Zn^2+^ concentrations, lower than that found in serum (15–25 μmol/l) [12,13], can affect free insulin concentrations, thereby improving the self-association of monomers to form Zn^2+^-insulin hexamers [5]. Furthermore, Zn^2+^ concentrations in the secretory granule have been estimated to be approximately 20 mmol/l [14]. Consequently, high concentrations of Zn^2+^ ions are likely to be produced locally in the interstitial space and in the capillaries of the pancreatic-islet core. In this environment of high Zn^2+^ concentration, it may be possible that a major fraction of Zn^2+^-insulin does not dissociate per se into monomers after exocytosis. It may be thus conceivable that Zn^2+^ chelation could facilitate hexamer dissociation. We postulate that blood albumin could play a significant role in this respect. Albumin binds to Zn^2+^ with high affinity [15] and is the main transport protein of Zn^2+^ in blood [16]. Additionally, the islet is a notably well-vascularized tissue, and albumin arrives close to the places of insulin exocytosis. While any intracellular space in the islet may be considered virtual, the intravascular space represents 20% of islet architecture. Furthermore, it is known that in certain cases, albumin penetrates into the extracellular space between the endocrine cells [17]. Finally, while electrical activity and intracellular calcium measurements in β-cells can be performed in free albumin solutions [18–20], secretion experiments require the presence of albumin in the medium [19,20]. Thus, albumin seems to affect insulin secretion in steps downstream of the calcium-dependent exocytotic process. The fact reported in this study, that both albumin and Zn^2+^ chelation allow insulin to be measured by radioimmunoassay, leads us to hypothesize a physiological role for this plasma protein in hexamer dissociation and the subsequent biological activity of the hormone.

In the present study, we analyze the effect of the Zn^2+^ binding properties of serum albumin on insulin hexamer dissociation and its relevance for insulin determination, secretion and pharmacokinetics.

## Materials and methods

### Solutions and reagents

All of the insulin release experiments were carried out at 37°C with fresh modified Krebs-Ringer bicarbonate (KRB) buffer containing (in mmol/l): 120 NaCl, 5 KCl, 25 NaHCO_3_, 1.1 MgCl_2_, 2.5 CaCl_2_. For islet isolation and insulin dilution KRB buffer containing (in mmol/l): 115 NaCl, 5 KCl, 10 NaHCO_3_, 1.1 MgCl_2_, 1.2 NaH_2_PO_4_ and 25 acid Hepes was used. All of the previously mentioned solutions were prewarmed at 37°C and continuously gassed with a mixture of O_2_ (95%) and CO_2_ (5%) for a final pH of 7.4. The medium used for islet culture was RPMI 1640 supplemented with 10% fetal calf serum, 100 IU/ml penicillin, 0.1 mg/ml streptomycin, and 11 mmol/l glucose. For insulin tolerance test (ITT), 4 μmol/l insulin stocks, of Zn^2+^-free or Zn^2+^-insulin, with or without 3% bovine serum albumin (BSA) were prepared and kept at 37°C for 20 min before injection. Human recombinant free insulin (Humulin Regular) and Zn^2+^-insulin (Ultralent Humulin) were from Lilly (Indianapolis, IN, USA). BSA, 2-carboxy-2’-hydroxy-5’sulfoformazyl-benzene (Zincon) and 2,2’:6’,2”-terpypridine (Terpy) were from (Sigma, St. Louis, MO, USA).

### Islet isolation

Islets from adult (8–10 weeks old) male Swiss albino mice (Charles River, France) were isolated as previously described [21]. Briefly, after pancreatic digestion with collagenase (Collagenase A; Boehringer Mannheim, Germany) in a stationary bath at 37°C, islets were separated by centrifugation and hand-picked under a stereomicroscope. Once isolated, the islets were then centrifuged and resuspended in culture medium, kept at 37°C in a humidified atmosphere of 95% O_2_, 5% CO_2_ and used between 2–3 h of culture. Mice were sacrificed by cervical dislocation.

### Insulin tolerance test (ITT)

Male Wistar rats (8–9 weeks old) (Charles River, France) were allowed free access to standard food and water ad libitum until experiments were performed. For experimental procedures rats were fasted for 5–6 h. Anesthesia was performed by intraperitoneal injection of sodium pentobarbital (60 mg/kg body weight) and the experiments were carried out after loss of corneal and pedal reflexes. The previously mentioned insulin stocks (4 μmol/l) were subcutaneously injected at 0.5 ml/kg body weight into the rats' hind back, and blood samples were collected from the tail at 0, 10, 20, 30, 40 and 60 min for measurement of serum glucose and insulin. Rats were sacrificed by terminal anesthesia with sodium pentobarbital.

This study was conducted in strict accordance with the recommendations in the Guide for the Care and Use of Laboratory Animals of the National Institutes of Health. The protocol was approved by the Committee on the Ethics of Animal Experiments of the Andalusian Center of Molecular Biology and Regenerative Medicine (Protocol number: 06-10-14-138). All surgery was performed under sodium pentobarbital anesthesia, and all efforts were made to minimize suffering.

### Insulin release experiments

Insulin measurements were performed as previously described [20]. For static incubation, islets were placed in groups of 3, for 30 min at 37°C, in 1 ml of KRB buffer with or without 2.5% BSA plus the different glucose concentrations (2.8 and 22.2 mmol/l). For islet perifusion experiments, batches of 10 large-size islets (308 ± 49 μm in diameter) or 40 small-size islets (56 ± 11 μm in diameter) were placed in a 100 μl chamber and perifused with KRB buffer with or without 1 mmol/l Zincon plus 0.1% BSA and 2.8 mmol/l glucose at a flow rate of 1 ml/min. The pancreatic islets were first perifused in the presence of 2.8 mmol/l glucose for 30 min to reach a state of insulin release. Throughout the perfusion, effluent was continuously collected at 30-second intervals. The dead time was 2 min and has been corrected for in the figures. Both static incubation and perifused samples were kept at -20°C until insulin determination.

### Insulin determination

Insulin was assayed by radioimmunoassay (RIA) using a kit from DPC (Los Angeles, CA, USA). The RIA included polypropylene tubes coated with antibodies to human recombinant insulin and was carried out following these steps: i) addition of samples to the antibody-coated tubes; ii) addition of freshly labeled ^125^I-(TYR A19)-human insulin and iii) incubation for 18–24 h at 4°C. Rat insulin was provided by the above-mentioned kit. Human recombinant free insulin was from Lilly. Both types of insulin were used to prepare standard curves. The standard curves and problems were run in duplicate.

### Statistical analysis

Statistical analysis was performed using GraphPad Prism 5.0 software (GraphPad Software Inc., La Jolla, Ca, USA). The results were expressed as the mean and standard error of the mean (± SEM). Differences among groups were determined by ANOVA followed by Tukey′s multiple comparison test. Normal distribution analysis before ANOVA analysis was performed. The unpaired Student′s t test (two tailed) was also used. A.*P*< 0.05 was considered to be significant. Figures were generated with GraphPad Prism 5.0.

## Results

### Effect of serum albumin on insulin radioimmunoassay

To evaluate the potential of RIA technique to discern the different antigenic forms of insulin, Zn^2+^-insulin and Zn^2+^-free insulin were diluted in KRB buffer without BSA at the same known concentration and incubated with I^125^ insulin in antibody-coated tubes. The results were compared with standards prepared with the same commercial Zn^2+^-free insulin.

As shown in Fig 1.

**Fig 1:**
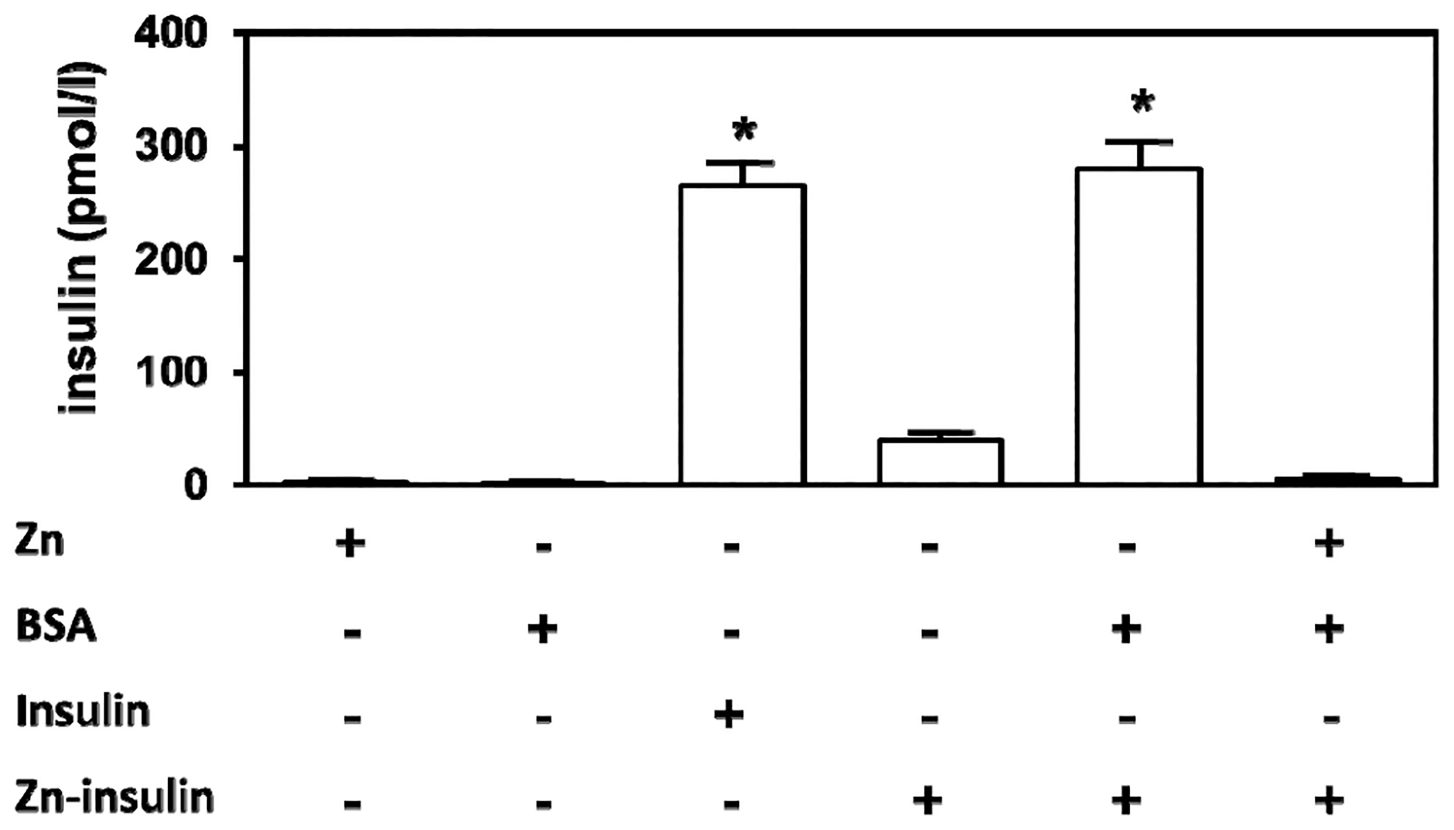
T. Effect of serum albumin on insulin determination by radioimmunoassay. Insulin concentration was measured in different KRB buffer solutions with 2 mmol/l ZnCl_2_ (Zn), 3% BSA (BSA), 250 mmol/l Zn^2+^-free insulin (insulin) and 250 mmol/l Zn^2+^-insulin (Zn-insulin). Values are expressed as the mean ± SEM of 6 experiments. *P<0.01 compared with the other conditions.

3% BSA and 2 mmol/l Zn^2+^ had no effect on the RIA technique when added alone. Although all insulin solutions had been prepared at the same concentration (250 pmol/l), Zn^2+^-insulin reacted with the antibody to a much lesser degree than Zn^2+^-free insulin (*P<0.01). While the Zn^2+^-free-insulin concentration determined was close to the expected concentration (262.9 ± 45.5 pmol/l), RIA failed to determine the accurate Zn^2+^-insulin complex concentration (39.1 ± 19.6 pmol/l). However, no significant differences were found between Zn^2+^ free insulin and Zn^2+^-insulin plus 3% BSA (262.9 ± 45.5 and 279.9 ± 60.8 pmol/l respectively), most likely due to chelation of the Zn^2+^ by serum albumin and the subsequent dissociation of Zn^2+^-insulin into Zn^2+^-free insulin. Thus, when 2 mmol/l ZnCl_2_ was added into BSA-supplemented KRB buffer, the albumin-induced enhancement of insulin detection was inhibited and the insulin determination was 5.1 ± 5.0 pmol/l, probably due to saturation of the albumin Zn^2+^-binding sites.

### Effect of serum albumin on insulin secreted in static incubations

**Fig 2:**
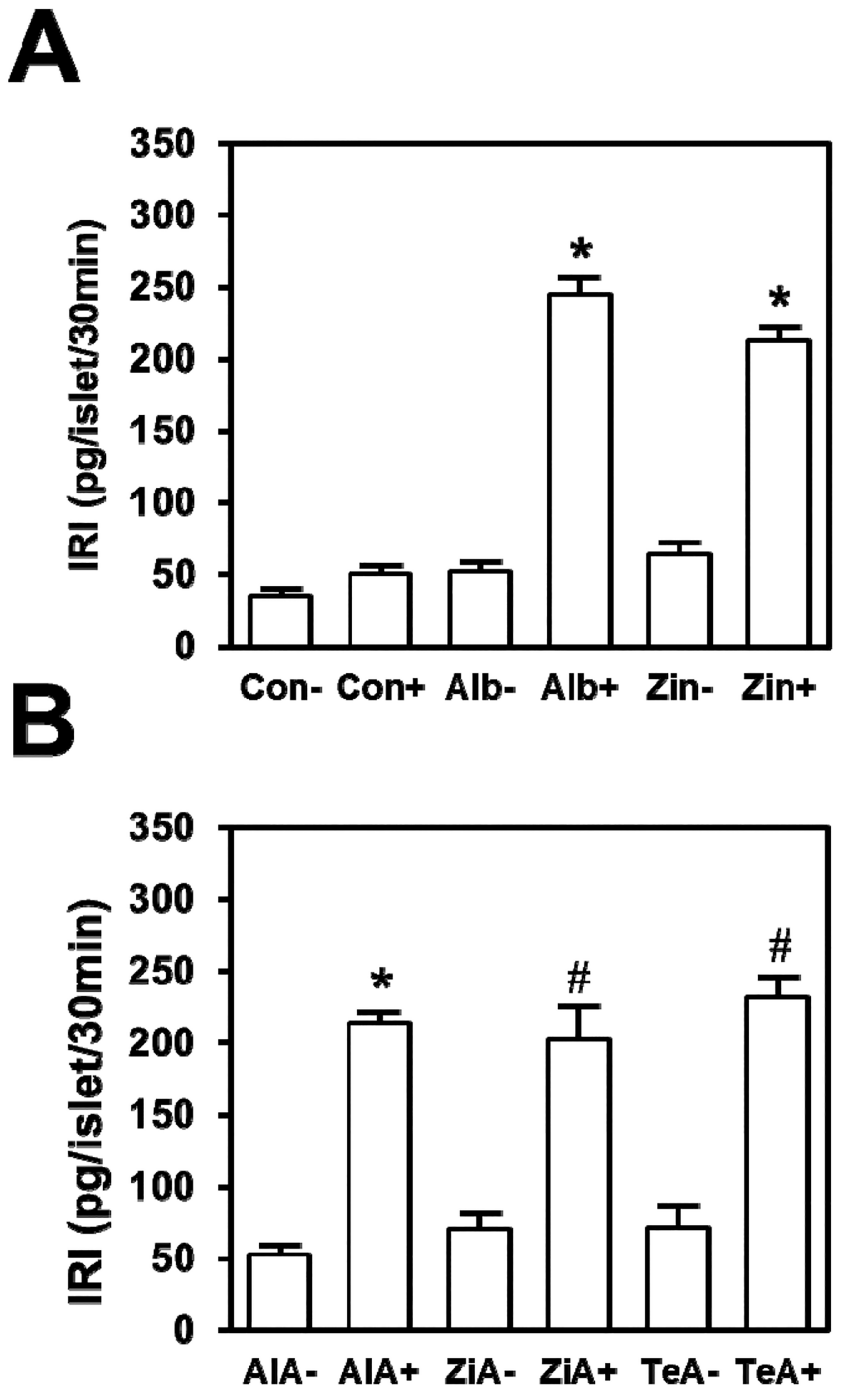
Effects of serum albumin and Zn^2+^ chelators on glucose-induced insulin secretion. Islets were incubated with KRB buffer supplemented with 2.8 (-) or 22.2 mmol/l (+) glucose. (A) Incubations were carried out with KRB buffer, in the absence of BSA and Zn^2+^ chelators (Con), plus 3% BSA (Alb) or 1 mmol/l Zincon (Zin). (B) Islets were incubated in KRB buffer alone and 3% BSA (AlA), 1 mmol/l Zincon (ZiA) or 10 mmol/l Terpy (TeA). Values are expressed as the mean ± SEM of 6 experiments. *P<0.01 compared with the other conditions. ^#^*P*<0.05 when ZiA+ and TeA+ were compared with ZiA- and TeA-, respectively.

Fig 2 shows effects of BSA and a Zn^2+^ chelator on insulin secreted in static incubation. When batches of 3 islets were incubated without BSA, no significant differences were found between the insulin detected after incubation of islets in 2.8 mmol/l glucose (35.9 ± 5.3 pg/islet/30 min) and stimulation with 22.2 mmol/l glucose (50.1 ± 10.5 pg/islet/30 min) (Fig 2A). However, the insulin detected in incubations with 3% BSA and 22.2 mmol/l glucose was 4.7-fold above that seen with BSA and 2.8 mmol/l glucose (245.4 ± 22.8 and 51.7 ± 11.7 pg/islet/30 min respectively, *P<0.01). Similar differences were obtained in islets incubated with 1 mmol/l Zincon when incubations at 22.2 and 2.8 mmol/l glucose were compared (212.4 ± 18.5 and 63.9 ± 16.4 pg/islet/30 min respectively, *P<0.01), suggesting that BSA affects insulin detection by its capacity to bind Zn^2+^.

To investigate if albumin was affecting insulin detection rather than insulin secretion, BSA was added into KRB buffer after incubation, when islets had been removed, but before insulin determination with RIA. The data are shown in Fig 2B. As in the case previously described with BSA during the incubation, when BSA was added after islets were removed, the insulin detected from experiments in 22.2 mmol/l glucose was 4-fold above that detected in 2.8 mmol/l glucose (214.3 15.4 and 52.4 10.8 respectively, *P<0.01). Additionally, no significant difference was found when comparing results obtained from islets incubated in 22.2 mmol/l glucose plus BSA (Fig 2A) with those obtained with the same glucose concentration but with BSA added into the buffer after incubation (Fig 2B) (245.4 ± 22.8 and 214.3 ± 15.4 pg/islet/30 min, respectively). Thus, serum albumin seems to affect insulin determination and not insulin secretion mechanisms. On the other hand, two Zn^2+^ chelators mimicked the effect of BSA on insulin determination. When Zincon was added into the effluent collected after incubation, the insulin detected from experiments with 22.2 mmol/l glucose was approximately 3-fold that detected from the control with 2.8 mmol/l (202.6 ± 53.3 and 71.0 ± 22.9 respectively, ^#^*P*<0.05). Additionally, Terpy showed similar effects (Fig 2B) (230.1 ± 30.9 and 71.6 ± 32.1 with 22 and 2.8 mmol/l glucose respectively, ^#^*P*<0.05). These results suggest that Zn^2+^ chelation by BSA facilitates Zn^2+^-insulin dissociation into monomers of Zn^2+^-free insulin after secretion, consequently monomeric insulin is detected with the RIA technique when BSA is absent; the predominant insulin form is Zn^2+^-insulin, which cannot be correctly determined (Fig 1).

### Effect of serum albumin on insulin-induced decrease in plasma glucose levels and subcutaneous insulin absorption

**Fig 3:**
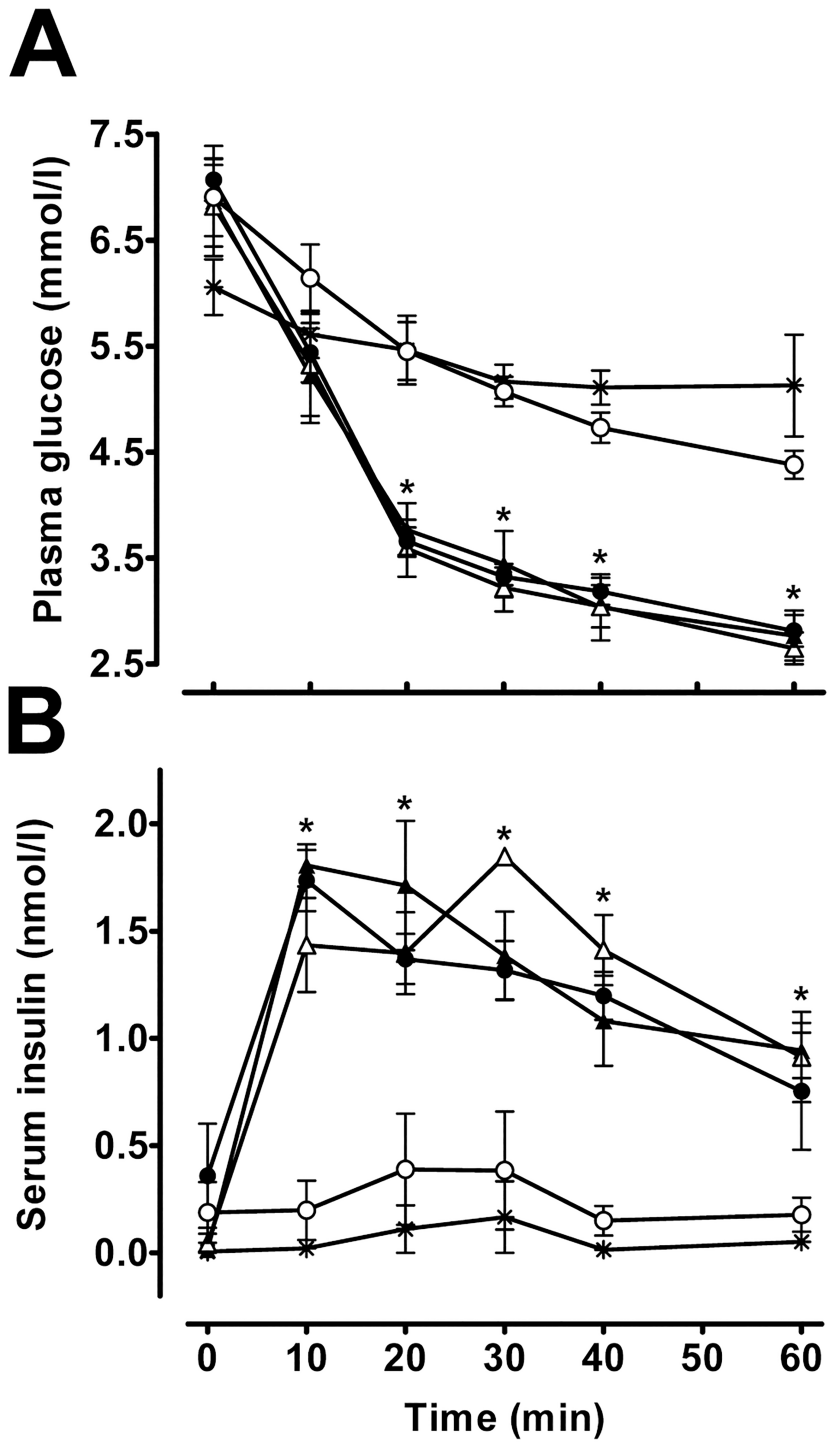
Effect of serum albumin on insulin pharmacokinetics. Rat plasma glucose levels (A) and serum insulin levels (B) after injection of Zn^2+^-free insulin (Δ), Zn^2+^-free insulin plus 3% BSA (▲), Zn^2+^-insulin (○), Zn^2+^-insulin plus 3% BSA (●) and Zn^2+^-insulin plus 2 mmol/l ZnCl_2_ and 3% BSA (*). Values are expressed as the mean ± SEM of 6 experiments for Fig 3A and 4 experiments for fig 3B. *P<0.01 compared with Zn^2+^-insulin and Zn^2+^-insulin plus 2 mmol/l ZnCl_2_ and 3% BSA.

It is established that Zn^2+^-insulin has different pharmacokinetic characteristics compared with Zn^2+^-free insulin. In fact, insulin has frequently been mixed with an excess of Zn^2+^ to obtain prolonged-action insulin preparations. To investigate the effects of serum albumin on Zn^2+^-insulin-mediated changes in plasma glucose levels, ITT was performed in anesthetized rats using different solutions of insulin (Zn^2+^-free insulin, Zn^2+^-free insulin plus BSA, Zn^2+^-insulin, Zn^2+^-insulin plus BSA and Zn^2+^-insulin plus 2 mmol/l ZnCl_2_ plus BSA) in KRB buffer. All of the solutions were preincubated at 37°C for 20 min before injection. The results are shown in Fig 3.

In Fig 3A a significant decrease (*P<0.01) in plasma glucose values was observed 20 min after Zn^2+^ free insulins and Zn^2+^-insulin plus BSA injections compared with the rest of the groups. A few minutes after injection of free insulin, a fall in plasma glucose levels was evident (6.8 ± 0.4 *vs*. 3.5 ± 0.2 mmol/l glucose at 0 and 20 min respectively, *P<0.01). Animals injected with Zn^2+^-insulin showed non-significant differences between their basal plasma glucose level measured at 0 min and that obtained at 20 min after injection (6.9 ± 0.4 and 4.8 ± 0.2 mmol/l glucose, respectively) (Fig 3A). However, injection of Zn^2+^-insulin plus BSA solution had an effect on plasma glucose levels similar to that of free insulin (7.0 ± 0.2 and 3.6 ± 0.1 mmol/l glucose at 0 and 20 min respectively, *P<0.01). Most likely, this decrease in plasma glucose levels was caused by Zn^2+^-insulin dissociation into Zn^2+^-free insulin due to BSA. The effects of free insulin and free insulin plus BSA on plasma glucose levels were also similar. Thus, as expected, BSA had no effect on Zn^2+^-free insulin. When Zn^2+^-insulin plus Zn^2+^-BSA was injected, its effects on plasma glucose levels were similar to those of Zn^2+^-insulin alone (6.0 ± 0.3 and 5.4 ± 0.4 mmol/l glucose at 0 and 20 min respectively). A very plausible explanation for this result is that the Zn^2+^ ion inhibited the effect of BSA on insulin-induced decrease of plasma glucose levels due to BSA Zn^2+^-binding site saturation.

Numerous lines of evidence provide strong support for the effects of hexamer aggregation on subcutaneous insulin absorption [22]. To determine whether BSA's effect on insulin-induced plasma glucose level changes was due to changes in insulin absorption kinetics, serum insulin levels were measured after injection of the previously mentioned solutions (Fig 3B). At 10 min after free insulin injection, serum insulin levels increased 32-fold compared to basal (0.04 ± 0.05 and 1.4 ± 0.2 nmol/l insulin at 0 and 10 min after injection respectively, *P<0.01) reaching a plateau (Fig 3B). Similar results were obtained after injection of free insulin plus BSA (0.01 ± 0.01 and 1.8 ± 0.1 nmol/l insulin at 0 and 10 min after injection, respectively, *P<0.01) and Zn^2+^-insulin plus BSA (0.6 ± 0.4 and 1.5 ± 0.1 nmol/l insulin at 0 and 10 min after injection respectively, *P<0.01). Otherwise, rats treated with Zn^2+^-insulin and Zn^2+^-insulin plus Zn^2+^-BSA solutions showed no significant increase of serum insulin levels at 10 min compared to basal levels (Fig 3B). In addition, Zn^2+^ free insulins and Zn^2+^-insulin plus BSA injections induced a significant increase (*P<0.01) of serum insulin levels compared with Zn^2+^-insulin and Zn^2+^-insulin plus Zn^2+^-BSA solutions. All of these results were consistent with the previously described effects on plasma glucose levels.

### Effect of a Zn^2+^ chelator on K^+^-induced insulin secretion in perifused islets of different sizes

In contrast with normal blood flow, in perifusion experiments the access of nutrients and release of insulin from islets are governed by diffusion laws. The isolation process causes a disruption of islets' vascular connections. Furthermore, pancreatic β-cells are densely packed at the core of islets. Consequently, the insulin secreted into extracellular space from β-cells, in isolated islets, must diffuse across the interstitial fluid to the islet surface before it is released to the surrounding medium. Thus, it is expected that isolated islets will show a different pattern of insulin release depending on the size of the islet. In this regard, insulin released from small islets would arrive faster to the surface than that released from large islets. Furthermore, the larger size of hexameric insulin probably lowers the diffusion rate through tissue. In fact, molecular size alters both the rate of diffusion through tissue as well as transport across the capillary membrane [23].

**Fig 4:**
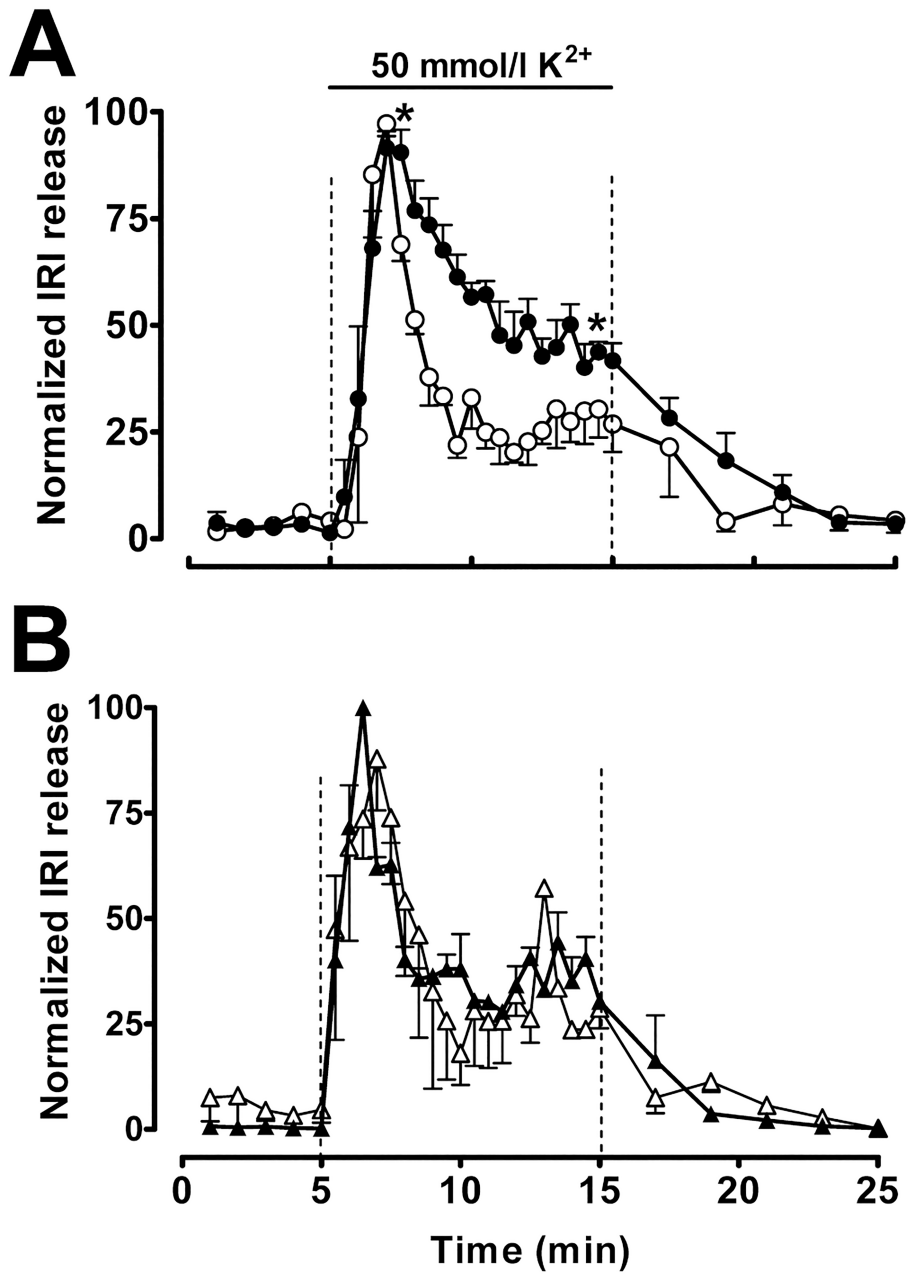
Effect of Zn^2+^ chelator on insulin secretion pattern. (A) Large (●) and small (○) islets were perifused with KRB buffer plus 0.1% BSA and 2.8 mmol/l glucose plus 50 mmol/l K^+^ as indicated by the bars. (B) Large (▲) and small (Δ) islets were perifused as previously mentioned but 1 mmol/l Zincon was added to the solution. Values are expressed as the mean ± SEM of 4 experiments. Data have been normalized to minimum and maximum values to compare insulin secretory patterns. *P<0.01 compared with small islets between minutes 7 and 15.

The pattern of high K^+^-induced insulin secretion from large (308 ± 49 μm) and small (56 ± 11 μm) islets is shown in Fig 4.

Perifused small-size islets responded to a stepwise increase in extracellular K^+^ with a clear-biphasic rise of insulin secretion, consisting of a 4 min transient burst followed by a plateau that lasted as long as the high K^+^ stimulus (Fig 4A). However, in perifused large-size islets, high K^+^ levels induced the same rapid increase of insulin release followed by a slow and steady fall in the insulin secreted (Fig 4A). If diffusion depends on hexamer size compared to monomer, a Zn^2+^ chelator will accelerate the rate. This is shown in Fig 4B. When zincon was added to the perifusion medium, the dynamics of high K^+^-induced insulin release from both large and small islets followed a similar well-marked biphasic pattern (Fig 4B). Zincon modified the pattern of high K^+^-induced insulin secretion from large islets, but no significant effect was found on the dynamics of insulin release from small islets (Fig 4B) compared with islets perifused without the Zn^2+^ chelator (Fig 4A).

## Discussion

### Serum albumin alters insulin measurements but not insulin release

As shown in Fig 1, while Zn^2+^-free insulin concentrations were determined with significant accuracy using an immunological assay, we failed to correctly determine Zn^2+^-insulin concentration in the absence of a Zn^2+^ chelator. This finding demonstrated that insulin immunoassay techniques efficiently recognize insulin monomer but not Zn^2+^-insulin polymer. Provided that an important part of the monomer of insulin is located at the hexamer-forming surface of the molecule [24], a highly significant fraction of the polyclonal antibodies against monomeric insulin used in the insulin immunoassay techniques are most likely incapable of recognizing the antigenic form of hexameric Zn^2+^-insulin. In agreement with this hypothesis, it has been suggested that Zn^2+^-insulin and Zn^2+^-free insulin are different antigenic forms and this can alter the insulin concentrations determined with immunological assays [25,26]. On the other hand, Fig 1 shows that when serum albumin is added, Zn^2+^-insulin can be determined at its actual concentration. Previous works have shown that serum albumin has a strong binding site for Zn^2+^ [15]. Furthermore, it is well established that at pH 7.3, Zn^2+^ ion is essential to containing the repulsion of the carboxylic acids of the six GluB13 residues packed closely together at the center of the insulin hexamer. When Zn^2+^ disappears, the strong mutual repulsion of these residues can cause the hexamer to disintegrate into monomers [11]. Thus, it is likely that albumin competes with insulin for Zn^2+^ and facilitates Zn^2+^-insulin disintegration into monomeric Zn^2+^-free insulin, thereby releasing the antigens hidden in the hexameric structure and allowing antibodies against monomeric insulin to bind with the monomers. As a matter of fact, when albumin's Zn^2+^ binding site was saturated with a high Zn^2+^ concentration, albumin did not improve the Zn^2+^-insulin determination as expected if albumin's effect was mediated by its Zn^2+^ binding properties (Fig 1). In contrast with their clear effects on Zn^2+^-insulin hexamers, albumin and Zn^2+^ chelators do not modify insulin release and proper glucose-induced insulin release is observed (Fig 2). Any direct effect of albumin or Zn^2+^ chelators on the insulin secretory machinery may be negligible since the absence of both albumin and Zn^2+^ chelators during the secretion process does not affect the measured insulin provided they are present during the immunological assay (Fig 2).

**Fig 5:**
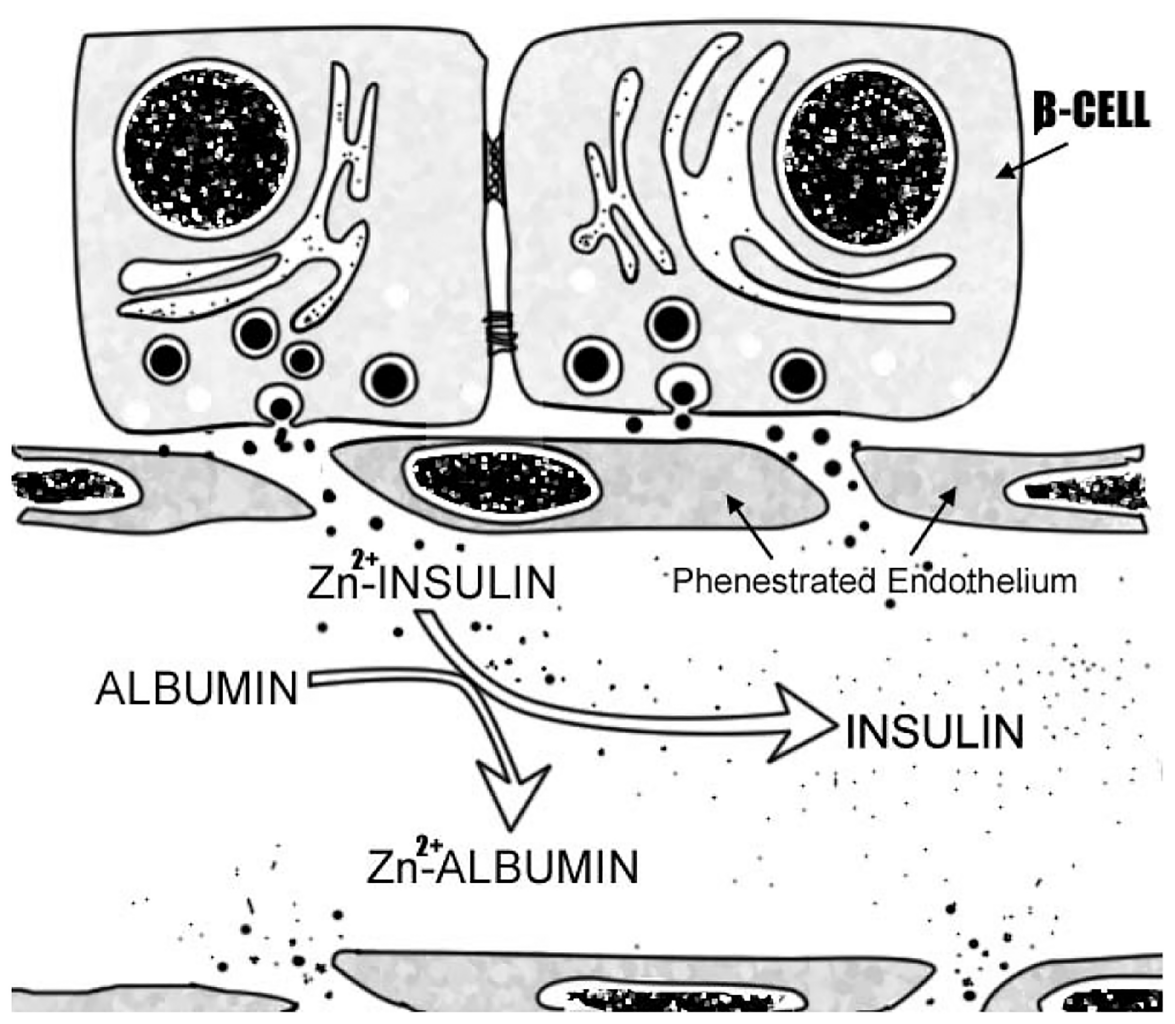
Serum albumin dissociates hexameric Zn^2+^-insulin into active monomers. Serum albumin chelates Zn^2+^. In turn, the Zn^2+^ free concentration decreases. Thus, serum albumin improves hexameric Zn^2+^-insulin dissociation into active Zn^2+^-free insulin monomer.

The most plausible hypothesis is that serum albumin chelates Zn^2+^ from Zn^2+^-insulin hexamer reversibly dissociating it into insulin monomer (Fig 5).

### Serum albumin may alter Zn^2+^-insulin's pharmacokinetic characteristics

The ratio of serum insulin level increase after subcutaneous administration depends on insulin absorption from the tissue. Several different variables are implicated in the absorption rate of subcutaneously injected insulin, such as subcutaneous fat thickness, skin temperature [27] and, especially, insulin characteristics [9,28]. Thus, capillary diffusion capacity depends on the size of the insulin molecule. Insulin monomer (approximately 6000 kD) is absorbed rapidly, and the larger Zn^2+^-insulin hexamer (approximately 36000 kD) is absorbed at much lower rates [22]. Subsequently, plasma glucose levels decreased faster after subcutaneous injection of Zn^2+^-free insulin than after Zn^2+^-insulin administration (Fig 3A). At the same time, faster increases in blood insulin levels were achieved with Zn^2+^-free insulin (Fig 3B). These results can be explained by differences in the absorption ratio of each insulin form. Conversely, pre-incubation with serum albumin altered Zn^2+^-insulin's pharmacokinetics, and under this condition, Zn^2+^-insulin mimics the effects on plasma glucose levels and serum insulin levels obtained with Zn^2+^-free insulin. Furthermore, Zn^2+^-free insulin's action was unaffected by previous incubation with albumin, and the effect of serum albumin on Zn^2+^-insulin pharmacokinetics was abrogated by addition of Zn^2+^ (Fig 3). All these data strongly support the notion that serum albumin increases the ratio of Zn^2+^-insulin absorption, improving the dissociation of hexamers into lower-molecular-weight subunits. Thus, serum albumin alters the characteristics of insulin, transforming long-action Zn^2+^-insulin into short-action insulin.

### Zn^2+^ buffering affects Zn^2+^-insulin diffusion through interstitial fluid after secretion

Insulin hexamers diffuse through the interstitial fluid of the extracellular space at approximately half the rate of insulin monomers [9]. Consequently, hexamers may be arriving at the surface of islets more slowly than the monomer form. The presence of a Zn^2+^ chelator accelerated the intercellular conversion of hexamers into monomers, and then the difference in the pattern between large and small islets disappeared (Fig 4B). Most likely, the low-molecular-weight Zn^2+^-chelator diffuses into the islets and improves hexamer dissociation into lower molecular size subunits, which diffuse quickly. Consequently, the pattern of insulin release was less affected by the size of the islets. These results support the hypothesis that insulin is secreted as a hexamer and requires a Zn^2+^ chelator to dissociate into subunits. In addition, the data suggest that the islet vascular network may be relevant to ensuring albumin availability in the core of the islets and fast conversion of Zn^2+^-insulin into the easily diffusible monomer after exocytosis. In addition, the studies [29,30] indicates that oxygen tension and islet size are important factors that affect insulin release in isolated islets.

### Technical and methodological considerations

This study has demonstrated that albumin or a Zn^2+^ chelator improves Zn^2+^-insulin disintegration into subunits, which could affect insulin detection by immunological assays. Thus, insulin determination in *in vitro* insulin-release experiments is clearly sensitive to the presence of serum albumin in the solution since insulin seems to be released as a hexamer [5,11]. These findings may raise doubts regarding the correct interpretation of the results of several studies, in which some authors evaluate the potential of different techniques to discern hexameric insulin from the monomer form, using albumin in their protocols [25,31]. On the other hand, Zn^2+^ inhibition of insulin [20,32] and glucagon release [33] have been reported. In these studies, the addition of Zn^2+^ into the solution may be saturating albumin's Zn^2+^ binding sites. Thus, Zn^2+^-insulin does not dissociate into subunits and could affect insulin-induced insulin release [10] and insulin's effects on glucagon release [33]. The authors suggest that Zn^2+^ has a direct effect on secretion; however an indirect effect on Zn^2+^-insulin dissociation cannot be ruled out. Other experiments can also be affected by serum albumin. The results from experiments testing the effects of drugs and nutrients on insulin release may be altered by the presence of albumin in the medium, since albumin binds to them [34] and reduces the active free concentration of the specific tested compound. Additionally, commercial albumin can be bound to nutrients, ions or other serum substances, and this could affect the results of some specific experiments. For these and other reasons albumin could affect the correct interpretation of data. Thus, replacement of albumin with Zn^2+^ might seem warranted in order to avoid those problems.

Pancreatic islets are densely vascularized micro-organs. Islet processing for clinical transplantation disconnects these structures from the vascular tree, disrupts the access to oxygen and nutrients [35] and delays insulin diffusion to the surface. While this may be secondary for small islets, it could affect the regulatory role of diffusion.

Our data suggest that Zn^2+^ insulin may not be properly dissociated in the absence of albumin, which could lead to suboptimal function of the islets after transplantation.

In conclusion, although it has been proposed that under physiological conditions changes in pH, clearance of insulin concentration and Zn^2+^ dilution result in hexameric insulin dissociation into subunits [5,11], these results indicate that the primary factor in this process is the chelation of Zn^2+^ by serum albumin. Several considerations agree with this hypothesis. It has been shown that Zn^2+^ concentrations (5 μmol/l) lower than those found in serum (approximately 15–25 μmol/l) could reduce hexameric insulin dissociation into monomer [5]. In addition, the dissociation of Zn^2+^-insulin just after exocytosis may be regulated by albumin concentrations in the extracellular space. It has been reported that the albumin concentration in the interstitial fluid of islets increases in diabetic patients [17]. However, whether changes in local concentrations of albumin affect the autocrine and paracrine functions of insulin in the islet remains to be explored.

Furthermore, islets are structures profusely irrigated. They represent 1% of the total mass of the pancreas and receive 20% of the blood flow. Islets' β-cells are close to the fenestrated endothelium (Fig 5), building a functional unit that is lost in islet transplantation procedures [36,37]. In other organs, such as the lung, free Zn^2+^ induces endothelial cell contraction [38], a process that is augmented by nitric oxide [39,40]. This mechanism might also operate in pancreatic islets in hypoxic conditions in a process dependent on the generation of high levels of nitric oxide [41,42]. Whether or not this observation is mimicked by human islet endothelium under inflammatory conditions needs further exploration. Moreover, in larger human, islets insulin- Zn^2+^ hexamers will take longer to reach the systemic blood flow. This diffusion delay will slow the corresponding regulatory feedback between peripheral tissue glucose uptake and the subsequent decrease in glucose-induced insulin release. Our observations open new venues in integrative islet physiology, pathophysiology and transplantation.

## Supporting information

S1 File Original data from effect of serum albumin on insulin determination by radioimmunoassay. (XLSX)

S2 File Original data from effects of serum albumin and Zn^2+^ chelators on glucose-induced insulin secretion. (XLSX)

S3 File Original data from effect of serum albumin on insulin pharmacokinetics. (XLSX)

S4 File Original data from the effect of Zn^2+^ chelator on insulin secretion pattern. (XLSX)
